# Needle tract seeding following endoscopic ultrasound-guided fine-needle aspiration for pancreatic cancer: a report of two cases

**DOI:** 10.1186/s12957-019-1681-x

**Published:** 2019-08-05

**Authors:** Toshiki Matsui, Kenichiro Nishikawa, Hiroki Yukimoto, Koji Katsuta, Yoshihumi Nakamura, Shota Tanaka, Michiaki Oiwa, Hiroki Nakahashi, Yuta Shomi, Yuji Haruki, Kentaro Taniguchi, Makoto Shimomura, Shuji Isaji

**Affiliations:** 1 Department of Surgery, Matsusaka Municipal Hospital, 1550, Tonomachi, Matsusaka, Mie Japan; 2 Department of Gastroenterology, Matsusaka Municipal Hospital, 1550, Tonomachi, Matsusaka, Mie Japan; 3 Department of Pathology, Matsusaka Municipal Hospital, 1550, Tonomachi, Matsusaka, Mie Japan; 4 Department of Hepatobiliary Pancreatic and Transplant Surgery, Graduate School of Medicine, Mie University, Tsu, Japan

**Keywords:** Endoscopic ultrasound-guided fine-needle aspiration, Needle tract seeding, Pancreatic cancer, Surgical resection, Gastric wall metastasis

## Abstract

**Background:**

Endoscopic ultrasound-guided fine-needle aspiration (EUS-FNA) is a useful tool in pancreatic cancer diagnosis. However, the procedure itself may cause peritoneal dissemination and needle tract seeding at the puncture site. We herein report two cases of gastric wall metastasis due to needle tract seeding after EUS-FNA.

**Case presentation:**

Case 1: A 68-year-old woman was admitted to our hospital for persistent cough. Computed tomography (CT) scan revealed inflammatory changes in the left lung field, and incidentally, a 15-mm hypovascular mass was detected in the pancreatic body. She underwent EUS-FNA and was diagnosed as pancreatic adenocarcinoma. She underwent distal pancreatectomy with splenectomy; however, a small hard mass was observed in the posterior gastric wall during surgery. We performed partial gastrectomy, and the resected specimen was diagnosed as a needle tract seeding following EUS-FNA. She then underwent adjuvant chemotherapy with TS-1, but the pancreatic cancer showed recurrence 6 months after surgery. She died due to peritoneal dissemination 18 months after surgery.

Case 2: A 70-year-old man was incidentally detected with a pancreatic body mass on a CT scan as part of his follow-up for recurrence of basal cell carcinoma. He underwent EUS-FNA and was diagnosed as pancreatic adenocarcinoma. He had nodules in both lungs, and it was difficult to differentiate them from lung metastasis of pancreatic cancer. Therefore, he underwent neoadjuvant chemoradiotherapy, and thereafter, the lung nodules showed no changes; hence, he underwent distal pancreatectomy with splenectomy. During surgery, we observed a hard mass in the posterior gastric wall. We performed partial gastrectomy, and the resected specimen was diagnosed as needle tract seeding due to EUS-FNA. He underwent chemotherapy with TS-1, and he is still alive 18 months after surgery at the time of writing.

**Conclusion:**

For resectable pancreatic body or tail tumors, EUS-FNA should be carefully performed to prevent needle tract seeding and intraoperative as well as postoperative assessment for gastric wall metastasis is mandatory.

## Background

Endoscopic ultrasound-guided fine-needle aspiration (EUS-FNA) for pancreatic tumors has pooled sensitivity and specificity at 92% and 96%, respectively [1], and it is an indispensable procedure for pancreatic cancer diagnosis. The main complications associated with EUS-FNA for pancreatic tumors is bleeding, pancreatitis, and post-procedural pain, among others, but the incidence rate is as low as 1.03%; therefore, EUS-FNA is considered a safe procedure [2]. The incidence rate of peritoneal dissemination associated with puncture for pancreatic cancer was reported to be 16.3% after percutaneous puncture and 2.2% after EUS-FNA; the risk of peritoneal dissemination is lower in EUS-FNA than in percutaneous puncture [3].

Needle tract seeding is a phenomenon in which tumor cells are found in the puncture route, and it is considered a subtype of peritoneal dissemination recurrence [4]. To the best of our knowledge, however, only 18 cases (17 reports) of needle tract seeding associated with EUS-FNA for pancreatic cancer have been reported till date [4–20]. Therefore, it is necessary to accumulate a greater number of cases of needle tract seeding for a better understanding of the features. Herein, we reported two cases of needle tract seeding after EUS-FNA that were detected during surgery and diagnosed via partial gastrectomy.

## Case presentation

### Case 1

**Fig. 1:**
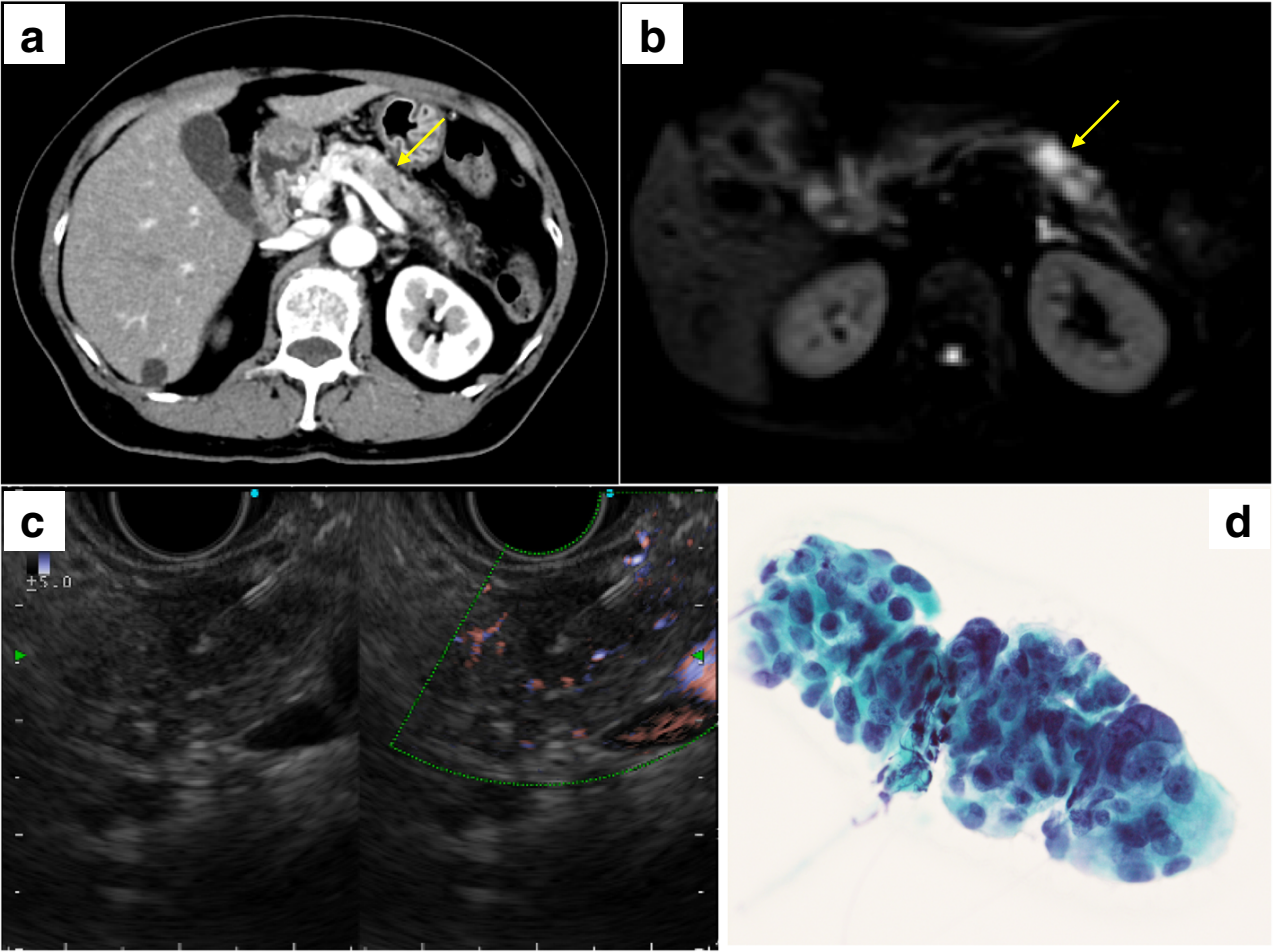
**a** Dynamic-enhanced computed tomography (portal phase) for case 1. A 15-mm hypovascular tumor was detected in the pancreatic body (arrow). **b** Diffusion-weighted magnetic resonance imaging. A hyperintense area can be observed in the pancreatic body tumor (arrow). **c** Endoscopic ultrasound-guided fine-needle aspiration (EUS-FNA). EUS-FNA was performed for the pancreatic tumor (4 punctures using 22 G, 19 G, 20 G, and 20 G needles) via the trans-gastric approach, without any complications. **d** Pathological findings of EUS-FNA. An adenocarcinoma can be observed (Papanicolaou staining)

**Fig. 2:**
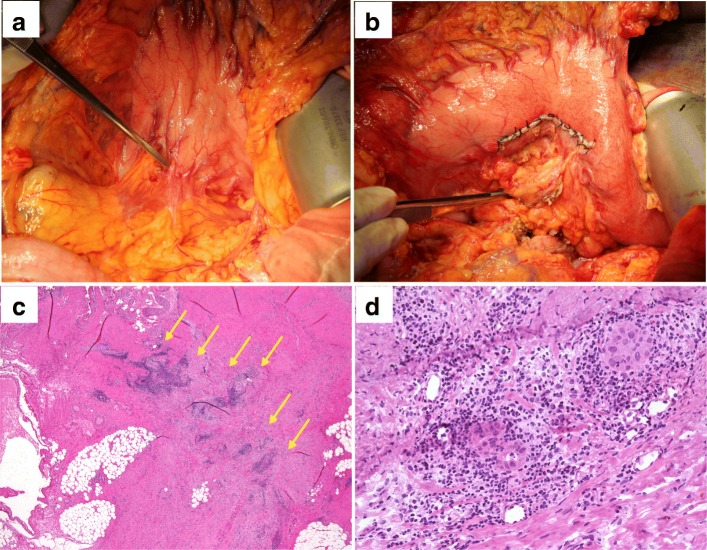
**a** Intraoperative findings for case 1. A small hard mass was detected in the posterior gastric wall, as indicated by the forceps. **b** Partial resection of the posterior gastric wall was performed. **c** Pathological findings. The specimen from the partially resected stomach showed that an adenocarcinoma was distributed linearly in the gastric muscle layer (arrow) (hematoxylin and eosin staining, loupe image). **d** Pathological findings. The findings of the gastric tumor were similar to those of the primary pancreatic cancer, indicating that gastric tumor was needle tract seeding from pancreatic cancer (hematoxylin and eosin staining)

A 68-year-old woman with no relevant medical or family history was admitted to our hospital because of a persistent cough. On admission, her abdomen was not tender and no mass was detected. Computed tomography (CT) scan revealed inflammatory signs in the left lung field along with incidental inflammatory findings around the pancreas, because of which pancreatitis was suspected. Dynamic-enhanced CT revealed a 15-mm hypovascular tumor in the pancreatic body (Fig. 1a), and inflammatory findings around the pancreas lead to the suspicion that concomitant pancreatitis is associated with pancreatic cancer. Laboratory data showed elevation of tumor marker levels (CA19-9, 44 U/ml; DUPAN-2, 1300 U/ml; Span-1, 33.0 U/ml). Diffusion-weighted magnetic resonance image revealed high-signal intensity in pancreatic body tumor (Fig. 1b). Endoscopic ultrasonography (EUS) revealed a 14.7 × 8.5 mm hypoechoic tumor in the pancreatic body, and the tumor did not contact to the superior mesenteric artery (SMA) and portal vein (PV). EUS-FNA for the pancreatic tumor was performed (4 punctures using 22 G, 19 G, 20 G, and 20 G needles) via the trans-gastric approach, and no complications were noted (Fig. 1c). Cytology revealed adenocarcinoma (Fig. 1d). Based on the imaging findings, she was diagnosed as resectable pancreatic body cancer. She underwent distal pancreatectomy with splenectomy. However, during surgery, we noticed a small hard mass in the posterior gastric wall (Fig. 2a), and thus, we performed partial gastrectomy (Fig. 2b). The pathological findings of the specimen from partially resected stomach revealed adenocarcinoma cells which were linearly distributed in the gastric muscle layer; these findings indicated that the gastric tumor was needle tract seeding from pancreatic cancer due to EUS-FNA (Fig. 2c, d). The time from EUS-FNA to the detection of the gastric wall metastasis due to needle tract seeding was 25 days. The pathological findings of the main pancreatic tumor resulted in a diagnosis of invasive ductal carcinoma, pT1, pN1 (No.8a, 11p), and pM0 pStageIIB (UICC). She then underwent adjuvant chemotherapy with TS-1, but a CT scan revealed peritoneal dissemination after 6 months. Therefore, the chemotherapy regimen was changed from TS-1 to gemcitabine (GEM) + nab-paclitaxel; however, her condition was gradually worsened and she died due to peritoneal dissemination of pancreatic cancer 18 months after surgery.

### Case2

**Fig. 3:**
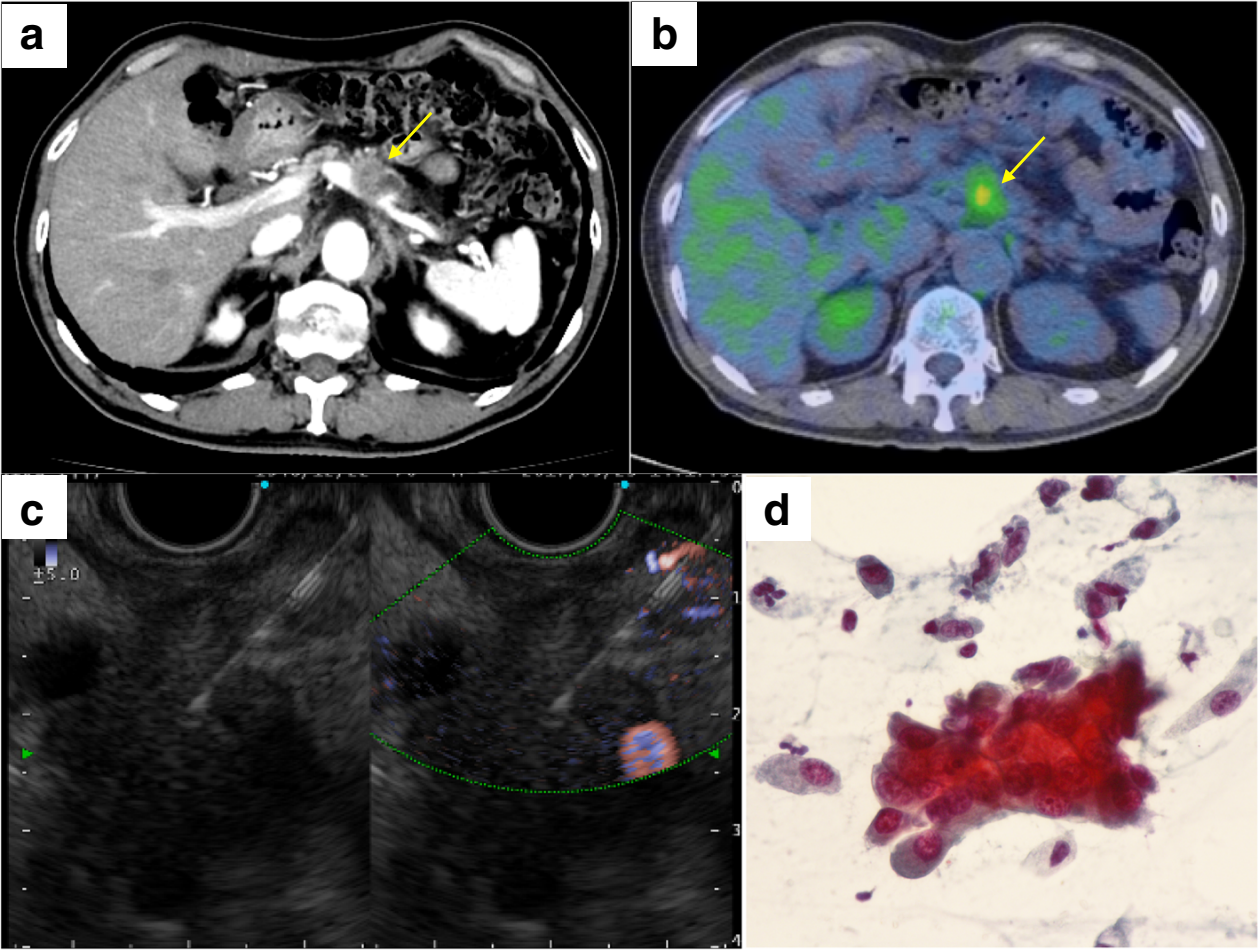
**a** Dynamic-enhanced CT (portal phase) for case 2. A 15-mm hypovascular tumor in the pancreatic body (arrow). **b** Positron emission tomography-CT (PET-CT) findings. Abnormal accumulation of fluorine-18-deoxyglucose (standardized uptake value of 3.74) can be observed in the pancreatic body (arrow). **c** EUS-FNA findings. EUS-FNA was performed for the pancreatic tumor (1 puncture using 22 G, needle) via the trans-gastric approach, without any complications. **d** Pathological findings. EUS-FNA revealed an adenocarcinoma (Papanicolaou staining)

**Fig. 4:**
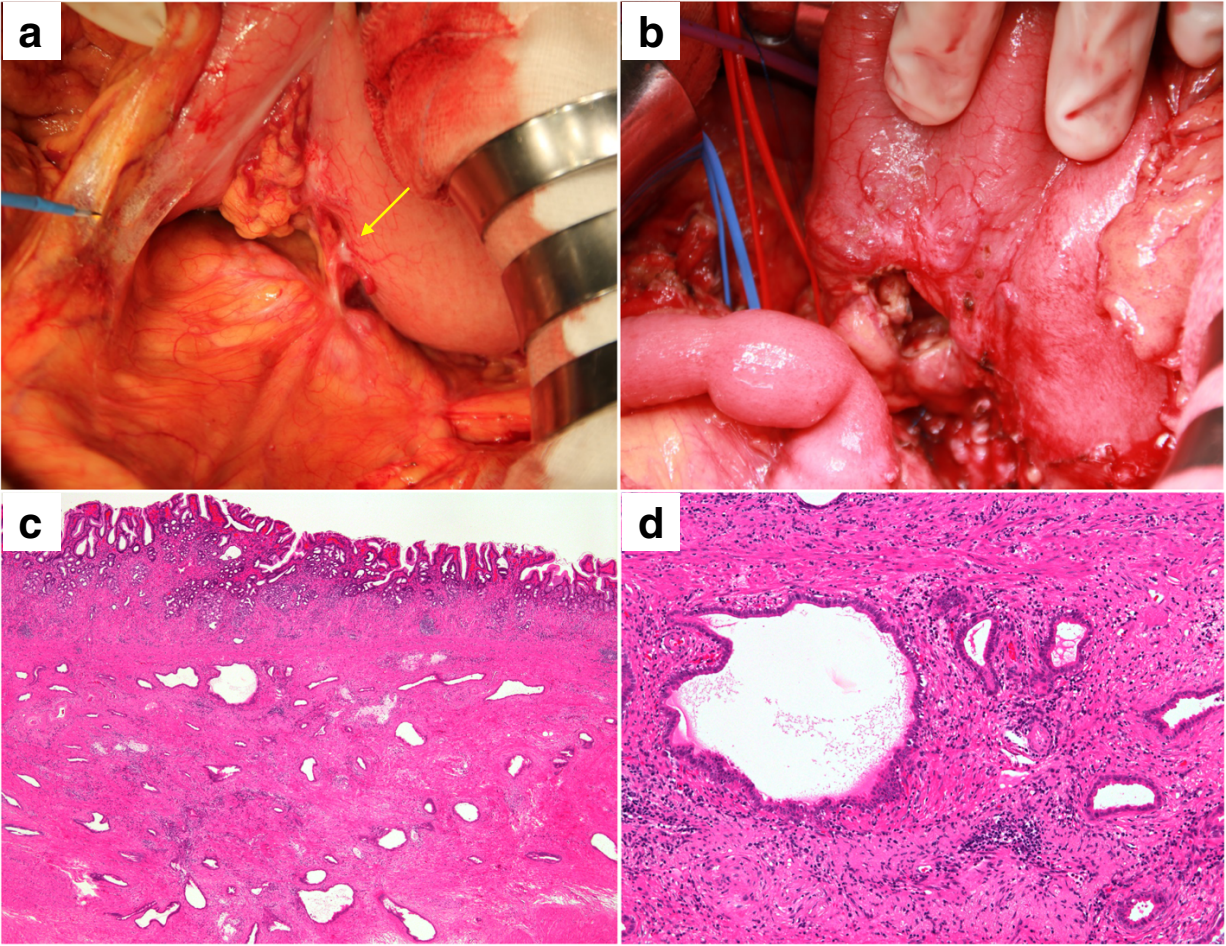
**a** Intraoperative findings for case 2. A small hard mass was detected in the posterior gastric wall (arrow). **b** Partial resection of the posterior gastric wall was performed. **c** Pathological findings. Many abnormal luminal structures (adenocarcinoma) were confirmed in the resected gastric muscle layer (hematoxylin and eosin staining, loupe image). **d** Pathological findings. The findings of gastric tumor were similar to those of the primary pancreatic cancer, indicating that gastric tumor was a recurrence due to needle tract seeding from pancreatic cancer (hematoxylin and eosin staining)

A 70-year-old man underwent CT scan as part of his follow-up for recurrence of basal cell carcinoma. Dynamic-enhanced CT scan incidentally revealed a 15-mm hypovascular mass 15 mm in size in the pancreatic body (Fig. 3a). He had no abdominal symptoms, and laboratory data showed no elevation in tumor marker levels. Positron emission tomography-CT (PET-CT) revealed abnormal accumulation of fluorine-18- deoxyglucose (FDG) in the pancreatic body, with a standardized uptake value of 3.74 (Fig. 3b); however, there was no abnormal accumulation of FDG in other parts of the body. EUS revealed a 15.2-mm hypoechoic tumor in the pancreatic body. Although his tumor was suspected to invade the splenic artery, the tumor did not invade the SMA or PV. EUS-FNA was performed (1 puncture using a 22 G needle) via the trans-gastric approach, and no complications occurred (Fig. 3c). Cytology revealed adenocarcinoma (Fig. 3d). He had small nodules in both the lungs, and it was difficult to differentiate them from lung metastasis of pancreatic cancer. Therefore, he underwent neoadjuvant chemoradiotherapy (50.4 Gy/28 Fr radiotherapy, and 2 cycles of chemotherapy: 600 mg/m^2^ GEM on days 8 and 22, and 60 mg/m^2^ TS-1 on days 1–21). After neoadjuvant chemoradiotherapy, his tumor marker levels were still within the normal ranges. The pancreatic tumor slightly shrunk, and small lung nodules showed no change. We suspected the lung nodules were not metastasis of the pancreatic cancer; therefore, he underwent radical antegrade modular pancreatosplenectomy procedure posterior (RAMPs posterior) [21]. During surgery, we noticed a small hard mass in the posterior gastric wall (Fig. 4a), for which we performed partial gastrectomy (Fig. 4b). The resected specimen was diagnosed as needle tract seeding following EUS-FNA (Fig. 4c, d). The time from EUS-FNA to the detection of the gastric wall metastasis due to needle tract seeding was 113 days. At the end of the surgery, a small nodule was found in the mesenterium of the small intestine. We resected it, and on pathological examination, it was diagnosed as peritoneal dissemination. Pathological findings resulted in a diagnosis of invasive ductal carcinoma, pT2, pN0, and pM1 pStageIV (UICC). He received chemotherapy with only TS-1, as GEM could not be used owing to allergic reactions observed during neoadjuvant chemoradiotherapy. His condition is stable even after 18 months after surgery at the time of writing.

## Discussion and conclusions

It has been reported that EUS-FNA for pancreatic tumor has pooled sensitivity and specificity, 92% and 96%, respectively [1]. The main complications of EUS-FNA for pancreas tumor is bleeding, pancreatitis, post-procedural pain, and so on, but the incidence rate is as low as 1.03%; therefore, it is considered a safe procedure [2]. Moreover, there was no significant difference in prognosis even when EUS-FNA was performed on the pancreatic body and tail cancer before surgery, and EUS-FNA is now an essential examination for the diagnosis of pancreatic tumors [22, 23]. However, in these reports, the type, stage, and resectability of pancreatic tumors were different and EUS-FNA has a risk of peritoneal dissemination, although its diagnosis due to FNA is difficult because pancreatic cancer itself often results in the development of peritoneal dissemination. Hence, the adverse effects of EUS-FNA may be ambiguous. In the future, the oncological safety of EUS-FNA should be reconsidered in limiting patients who undergo this procedure.

The first case of needle tract seeding after EUS-FNA in a patient with invasive ductal carcinoma derived from intraductal papillary mucinous neoplasm (IPMN) was first reported in 2003 [5], then in 2005, needle tract seeding after EUS-FNA was reported in a patient with a common type of pancreatic adenocarcinoma [6].In a search of the PubMed database and Ichushi (Japanese database) using the search term “[(endoscopic ultrasound fine-needle aspiration) OR (EUS-FNA) AND (pancreatic cancer) OR (pancreatic adenocarcinoma) AND (needle tract seeding) OR (seeding)],” till date, only 18 cases (17 reports) of needle tract seeding associated with EUS-FNA for pancreatic cancer have been reported, including invasive ductal carcinoma derived from intraductal papillary mucinous neoplasm (Table 1) [4–20]. Regarding the tumor site, all the tumors were located in the pancreatic body or pancreatic tail, except for a case with pancreatic head cancer who did not undergo surgery, and two cases where there was no description. This was probably because the puncture route is included in the resection range for pancreatic head cancer. In 3 of 18 cases, including our 2 cases, needle tract seeding was detected during surgery. Therefore, intraoperative assessment for gastric wall metastasis is important as well as postoperative assessment, and if surgeon suspects gastric wall metastasis intraoperatively, partial gastrectomy should be performed without hesitation. In these reported cases, the median period until the gastric wall metastasis after EUS-FNA is 21 months, but it occurred only 10 days in the shortest case [5]. As shown in our case 2, needle tract seeding after EUS-FNA cannot be controlled even after chemoradiotherapy.

**Table 1:** Reported cases of needle tract seeding after EUS-FNA for pancreatic tumor

Author		Year	Age	Sex	Location of pancreatic cancer	Tumor size	Frequency of puncture	EUS needle	Initial treatment	Stage	Discovery opportunity	Time to recurrence (months)	Recurrence tumor size	Treatment for needle tract seeding
1	Hirooka	2003	57	Male	Pancreatic body	20 mm	3	22 G	Distal pancreatectomy and partial gastrectomy	T1N0M0	Operative findings	1	Micro	Partial gastrectomy
2	Paquin	2005	65	Male	pancreatic tail	22 mm	5	22 G	Distal pancreatectomy	T1N0M0	CT	21	50 mm	Chemotherapy
3	Ahmed	2011	79	Male	Pancreatic body	Unknown	Several times	Unknown	Central pancreatectomy	T2N0M0	PET-CT	39	45 mm	Total gastrectomy
4	Chong	2011	55	Female	Pancreatic tail	27 mm	3	22 G	Distal pancreatectomy	T2N0M0	PET-CT	26	40 mm	No indication of surgery
5	Katanuma	2012	68	Female	Pancreatic body	20 mm	4	22 G	Distal pancreatectomy	T2N0M0	EGDS	22	Unknown	Unknown
6	Anderson	2013	51	Male	Pancreatic head	50 mm	Unknown	Unknown	Chemoradiation therapy	Unknown	EGDS/EUS-FNA	Unknown	10 mm	Unknown
7	Ngamruengphong	2013	66	Male	Pancreatic body/tail	Unknown	3	22 and 19 G	Subtotal pancreatectomy	Unknown	EGDS/EUS	27	Unknown	Unknown
8	Ngamruengphong	2013	77	Female	Pancreatic tail	40 mm	3	19 G	Distal pancreatectomy and partial gastrectomy	Unknown	EGD	26	Unknown	Unknown
9	Sakurada	2015	87	Female	Pancreatic body	25 mm	Unknown	22 G	Distal pancreatectomy	T2N0M0	Elevation of CA19-9	19	20 mm	Partial gastrectomy
10	Minaga	2015	64	Female	Pancreatic body	20 mm	3	22 G	Distal pancreatectomy	T3N0M0	Elevation of CA19-9	8	12 mm	Partial gastrectomy
11	Tomonari	2015	78	Male	Pancreatic body	20 mm	2	22 G	Distal pancreatectomy	T3N0M0	EGDS	28	32 mm	Subtotal gastrectomy
12	Kita	2016	68	Female	Pancreatic body	Unknown	2	22 G	Intensity-modulated radiation therapy	Unknown	PET-CT	4	Unknown	Unknown
13	Yamabe	2016	75	Male	Unknown	30 mm	Unknown	25 G	Chemotherapy	Unknown	CT/EUS-FNA	3	24 mm	Chemotherapy
14	Minaga	2016	72	Male	Pancreatic body	10 mm	Unknown	Unknown	Distal pancreatectomy	T1N0M0	EGDs/EUS	24	30 mm	Gastrectomy
15	Iida	2016	78	Female	Unknown	Unknown	3	22 G	Distal pancreatectomy	T3N0M0	EGDS/PET-CT	6	18 mm	Distal gastrectomy
16	Yamauchi	2016	67	Female	Pancreatic body	25 mm	1	19 G	Distal pancreatectomy	T3N0M0	EGDS/EUS-FNA	23	28 mm	Partial gastrectomy
17	Sakamoto	2018	50	Male	Pancreatic tail	38 mm	2	22 G	Distal pancreatectomy	T4N1M0	EGDS	24	20 mm	Partial gastrectomy
18	Matsumoto	2018	50	Male	Pancreatic body	35 mm	3	21 G	Distal pancreatectomy and partial gastrectomy	Unknown	CT/EUS	8	Unknown	Partial gastrectomy
19	Our case 1	2019	68	Female	Pancreatic body	15 mm	4	22, 19, 20, and 20 G	Distal pancreatectomy and partial gastrectomy	T1N1M0	Operative findings	1	Micro	Partial gastrectomy
20	Our case 2	2019	70	Male	Pancreatic body	34 mm	1	22 G	Distal pancreatectomy and partial gastrectomy	T3N0M1	Operative findings	4	Micro	Partial gastrectomy

*Abbreviations*: *CT* computed tomography, *PET-CT* positron emission tomography computed tomography, *EGDS* esophagogastroduodenoscopy, *EUS* endoscopic ultrasound, *EUS-FNA* endoscopic ultrasound-guided fine-needle aspiration

According to NCCN guidelines [24] and clinical practice guidelines for pancreatic cancer 2016 from the Japanese Pancreas Society guidelines [25], the treatment policy of pancreatic cancer varies according to the tumor resectability; surgery is the first treatment choice for resectable pancreatic cancer. For borderline resectable pancreatic cancer, it is a dominant opinion that neoadjuvant chemoradiotherapy is known to improve the prognosis, and for unresectable cases, chemotherapy is chosen. If we choose to perform chemotherapy for pancreatic cancer including preoperative treatment, it is necessary to differentiate it from other pancreatic tumors via EUS-FNA. However, whether EUS-FNA should be performed for all pancreatic tumors is controversial. Depending on the resectability and the localization of the tumor, it is necessary to consider the indications of EUS-FNA separately. For resectable pancreatic cancer that does not conflict with pancreatic cancer on the imaging studies, there may be a choice not to puncture the tumor. When EUS-FNA is performed for pancreatic body or tail cancer which is not included in the resection range, we should be aware of the risk of developing needle tract seeding in the gastric wall. In order to avoid needle tract seeding, biopsy needle with a covering sheath should be used [26]. Although our institution had already used a biopsy needle with a covering sheath, needle tract seeding unfortunately developed in these two cases. Therefore, the other factors such as technical problem should be considered. To prevent needle tract seeding as much as possible, we recommend to avoid unnecessary EUS-FNA for resectable pancreatic body or tail cancer, when up-front surgery is planned. Actually, when we consider the cost of EUS-FNA and the selection of operative procedure for the patients in whom up-front surgery is planned, EUS-FNA has few benefits because EUS-FNA by itself does not influence the selection of the operative procedure and is costful. If EUS-FNA is performed, intraoperative and postoperative assessment is essential for gastric wall metastasis due to needle tract seeding. According to the report by Yamauchi et al., if gastric wall metastasis due to needle tract seeding is detected early, partial gastrectomy can control the disease [4]. However, if the finding of gastric wall metastasis due to needle tract seeding is delayed, there is a report that lymph node metastasis has occurred [7]. In addition, there is a report of recurrence after partial gastrectomy for gastric wall metastasis due to needle tract seeding [18]; hence, post-operative assessment is important.

In conclusion, although EUS-FNA is a useful diagnostic tool, it may cause peritoneal dissemination and needle tract seeding at the puncture site. Therefore, physicians should decide its indication, especially for resectable pancreatic body or tail cancer, by taking the consideration of merit and demerit of EUS-FNA for each case.

## Data Availability

The datasets obtained during the current study are available from the corresponding author on reasonable request.
